# Analysis of Green Public Procurement of Works by Spanish Public Universities

**DOI:** 10.3390/ijerph15091888

**Published:** 2018-08-31

**Authors:** Jose Luis Fuentes-Bargues, Pablo Sebastian Ferrer-Gisbert, Mª. Carmen González-Cruz

**Affiliations:** GIDDP, Departamento de Proyectos de Ingeniería, Universitat Politècnica de València, Camino de Vera s/n, 46022 Valencia, Spain; pferrer@dpi.upv.es (P.S.F.-G.); mcgonzal@dpi.upv.es (Mª.C.G.-C.)

**Keywords:** green public procurement, environmental criteria, Spanish universities, works, tendering

## Abstract

Universities play an important role among public institutions because they initiate huge purchasing and contracting activities and contribute to sustainable development through education, research, and day-to-day operations. Existing studies on green public procurement (GPP) practices at Spanish universities focus on products and services. For this study, a total of 316 procedures were collected and analysed from the calls for tenders made by Spanish public universities between 2016 and 2017. The environmental criteria involved in the tenders and their weights were classified by subsector, geographical scope, and project budget. The results of this study show the use of environmental criteria in the works tendered by Spanish public universities is low (19.2%) in comparison with the results of other studies. It is therefore necessary to encourage GPP practices in the contracting process to comply with the environmental policies that universities have defined as part of their institutional policies.

## 1. Introduction

Public procurement accounts for approximately 10 to 15% of the gross domestic product (GDP) of developed countries [1,2,3], and in other countries, these values are even greater [4,5]. Public authorities are one of the main consumers of products, services, and works—and such authorities can play a crucial role in making consumption more sustainable [6,7].

In the World Summit on Sustainable Development (WSSD) held in Johannesburg in 2002, an implementation plan to support regional and national initiatives was created to accelerate the shift towards sustainable consumption and production (SCP) and de-link economic growth from environmental degradation. In June 2003, as a consequence of the Marrakech process, the Marrakech Task Forces were created to support the implementation of specific projects on specific sustainable consumption and production (SCP) themes. One of the seven task forces was led by Finland and entitled ‘Sustainable Buildings and Construction’, and its objectives included encouraging sustainable public procurement (SPP) [8].

SPP was defined by the United Nations as a procurement wherein an organization uses its buying power to signal preferences to the market with its choice of goods and services that meet sustainable criteria [9]. Within the public policies of SPP, environmental aspects have been increasingly considered [10] and have led to the concept of green public procurement (GPP).

GPP was defined by the European Commission as a: ‘process whereby public authorities seek to produce goods, services, and works with a reduced environmental impact through their life cycle when compared to goods, services, and works with the primary function that would otherwise be procured’ [11].

Green Public Procurement is supported by several international development policies and strategies, and is an important instrument that contributes to the achievement of economic and environmental objectives [12]. Administrations, as major consumers, can potentially orient production and consumption trends and encourage the demand of environmentally friendly products and services [13,14,15]. Although other authors, as for example Nikolau and Lizou [16], in their research on Greece and Cyprus during the economic crisis period have pointed out that GPP is not a suitable way to improve the adoption of environmental management practices due to the austerity measures and budgetary cuts. In any case, the interest in GPP from administrations, as well as researchers and academics, has increased significantly in recent years [17]. 

Universities are included within public administrations. They play an important role in the category of public institutions because they conduct huge purchasing and contracting activities [18,19] and contribute to sustainable development through education, research, and daily operations [20]. Several studies have been published about GPP practices at Spanish universities [19,21], but they are focused on products and/or services, rather than the works of construction and facilities. 

The practices of GPP in the construction sector must be studied for two main reasons: The high impact on the environment; and the fact that it is one of the most important sectors in terms of the amount of money spent and expertise required. The Worldwatch Institute’s State of the World 2012 states that the construction industry consumes more than one-third of global resources [22] and the Organization for Economic Co-operation and Development (OECD) states that this sector consumes around 25–40% of final energy consumption in the OECD [10]. In economic terms, the construction sector represents 9.3% European Union GDP and employs 7% of the European workforce [23].

Uttam et al. [10] showed that an analysis of the uptake of GPP can be performed from three approaches: Technical specifications; award criteria; and contract performance conditions. Technical specifications indicate the prerequisites to submit a tender, award criteria enable a comparison of the different tenders, while contract performance conditions are included in the contract to indicate how the contract must be performed. Testa et al. [24] define five phases of the tendering process when analysing environmental criteria: Subject matter; selection criteria; technical specifications; award criteria; and contract performance clauses. Subject matter and technical specifications are usually included in the design process of buildings, roads, or facilities—and it is difficult to determine if sustainability or environmental protection is a minimum (normative) condition, or a desire of the project owner. Also contract performance conditions are usually very general and common to all types of contracts—so the analysis of the award criteria, specifically, the analysis of the environmental criteria, is the most common method for establishing the current level of GPP practice [8,24,25].

The main objective of this study is to analyseanalyse GPP in the public works of Spanish universities, through an analysis of the use of environmental criteria. The results contribute to knowledge about GPP and can help rectors and/or managers of universities understand the current situation and better develop the use of environmental criteria. The paper is divided into seven sections. The first is the introduction on the topic. Section 2 describes research on GPP and the regulatory framework in the European Union and Spain, and describes the Spanish university structure. Section 3 develops the method and results are presented in Section 4. The results are compared with results from other GPP studies in Section 5. Finally, Section 6 presents the conclusions.

## 2. Background and Previous Findings

The current EU regulatory framework is Directive 2014/24/EU [26] on public procurement (repealing Directive 2004/18/EC) [27]. Both standards deal with environmental characteristics such as tendering criteria. In Spain, Directive 2004/18/EC was transposed into Act 30/2007 [28] (which evolved to become a refunded text in Royal Decree 3/2011 [29]), and Directive 2014/24/EU was transposed to Act 9/2017 [30], which went into effect in March 2018. Accordingly, relevant environmental criteria must contemplate contract requirements from an environmentally friendly point of view, such as the reduction of emissions or noise, reduced consumption of resources, etc. These criteria, as far as possible, should be measurable and not assessed by value judgments [8].

Many advances have been carried out on GPP in the last decade, and it has become one of the primary pillars in the environmental and procurement policies of the European Union [31,32,33] and around the world (USA [34], China [35], Hong Kong [36]), and including developing countries (for example, Malaysia [37], Vietnam [38]).

In the European Union, Bouwer et al. [39] identified in 2006 two groups regarding the use of GPP among the members of the European Union: The ‘Green 7’ (Austria, Denmark, Finland, Germany, United Kingdom, Holland, and Sweden); and the ‘Other 18’. In this research, the authors carried out an analysis from two approaches. Firstly, 865 responses from 8787 questionnaires were analyseanalysed; and secondly, 1000 tender documents in EU countries were processed. The results show approximately 67% of the respondents reported the use of environmental criteria in procurement, while only 36% of the tender documents reveal some environmental criteria. This finding, in which public authority staff exaggerate the implementation of GPP, was in concordance with other authors [24,25,40].

Nissinen et al. [41] studied a sample of calls for tenders from Denmark, Finland, and Sweden between the years 2003 and 2005 and classified the products according to the importance of the environmental aspects included in the public procurement process—high, medium and low. They also identified a ‘well-defined environmental criteria’ as one for which the purchasing authority has given the information on how a criterion must be fulfilled and verified.

In Sweden, Carlsson and Waara studied green procurement in 2004 with a sample of 558 public authorities. The results show that 15% of the 400 respondents stated that they always use environmental requirement, 46% usually do, 27% sometimes do, and 10% seldom or never [31]. The authors also identified several limitations on the impact on the implementation of GPP: Lack of administrative resources (including environmental know-how); high cost of green products within lean budgets; and the possible claims by unsuccessful bidders regarding the vagueness of environmental criteria.

Parikka-Alhola et al. [42] examined the use of GPP in Finland, Sweden, and Demark through tender calls in 2005. They found almost one-third of the tender calls contained environmental criteria, including environmental policy and environmental management systems requiring the fulfilment of eco-label criteria (chemical content, recycling, or reuse systems), packaging material, and noise. They also found that the weight of the environmental criteria was between 5–20% of the award criteria with an average weight for green criteria of 3.3%. 

In 2007, Michelsen et al. [43] studied the green procurement practices at a local and regional level in Norway using two questionnaires, one addressed to personnel of municipalities and counties, and another addressed to personnel of suppliers of Norwegian public authorities (many of them were members of the Confederation of Norwegian Enterprises). Results showed that GPP is significantly more established in large municipalities than in small ones because large municipalities have more resources for establishing a purchasing department which can generate knowledge and develop purchasing strategies.

In their econometric analysis, Testa et al. [44] showed that the size of public authorities and the level of awareness of the existing tools for supporting GPP have a positive and significant effect on the probability that they adopt GPP practices. In another survey of administrators at the municipalities of Tuscany (Italy), Testa et al. [7] concluded that it is necessary to develop successful strategies, well-trained personnel, and dispose of guidelines and tools for GPP. 

Many authors concluded that one of the main difficulties in the implementation of GPP is the vagueness and lack of clarity of the environmental criteria themselves [5,7,36], even Large et al. [45] claimed contracting authorities do not consider the inclusion of environmental criteria because of the difficulty of monitoring environmental conditions during the execution of the project.

Other authors have pointed out the importance of the environmental criteria within the awarding process, as for example Igarashi et al. [46], who pointed out in a study of information and communication technology (ICT) tenders in Norway, that the environmental criteria were the third most frequent award criteria after price and quality, but with a lower weight than all other award criteria.

### 2.1. Research on GPP Construction

Research and policies on GPP construction has not been given as much attention as products and services, but interest has been increasing in recent years [17].

A traditional trend in research on public procurement has been the development of methodologies for the selection of contractors. For example, Pastor et al. [47] developed a multi-criteria analytic hierarchy process-analytic network process (AHP-ANP) methodology for the selection of criteria in public contracts. Bendaña et al. [48] studied the implementation of neural networks and diffused techniques in the selection of contractors, and Moretti et al. [49] developed a method based on the weighted sum multi-criteria analysis to estimate environmental damage and choose the most environmentally-friendly solutions. 

The different authors proposed various environmental criteria in their methodologies, such as the environmental characteristics of the company, the environmental management system (EMS) of the company, and the environmental control of the project. 

In reference to the EMS, Lam et al. [50] made a study in China about GPP and one of their conclusions was that construction companies with environmental management systems (EMS) have similar attitudes towards green specifications as companies without EMS, and that simply encouraging EMS in the construction industry is insufficient to force the inclusion of green considerations. Testa et al. [7] concluded in their study on GPP in the municipalities of Tuscany (Italy) that a certified EMS provides a growing valued added factor to GPP practices and influences the percentage of green tenders. 

Regardless of the possible influence on the improvement of GPP practices, it is necessary to remember that from the point of view of a legal framework, at least in Spain, the possession of a certified EMS cannot be used as a criterion for the adjudication of the contract and can only be used as a criterion for the technical solvency of the bidder [51].

The European Commission published two documents in 2008 entitled ‘GPP training toolkit—Module 3: Purchasing recommendations—construction: Background product report’ [52] and ‘Construction: GPP product sheet’ [53] in which examples of green criteria for each stage of a tender are defined. The objective of these documents is to support public authorities in the implementation of GPP. Two levels of criteria were defined: Core criteria and comprehensive criteria. The first level does not increase the cost and complexity of the tendering criteria, while the second level does increase costs. 

The baselines for both criteria are: life cycle assessment (LCA); use of environmental product declarations (EPD) and their levels of CO_2_ emissions; use of materials with recycled and reused content; and the requirement to reduce emissions generated by the transport of heavy materials. These criteria are gradually being implemented and represent a branch of research at GPP. For example, Butt et al. [54] studied in 2015 the LCA for the green procurement of roads and claimed that the methodology was not integrated into the practice and that the boundaries of the LCA depended of the hierarchy of the decision level and the stage in the planning process, so increasing the level of consistency and transparency in pre-procurement and procurement phases. Similar conclusions were made by Lenferink et al. [55] in research on design-build-finance-maintain (DBFM) contracts in Dutch infrastructure projects.

The analysis of carbon footprint (CF) also can be used in GPP, as in the example developed by Alvarez and Rubio [56] in the urban waterfront and riverside of a 30-km stretch of the River Manzanares in Madrid (Spain). CF can act as a high stimulus for eco-innovation and reduced fuel consumption. 

Very little research has been done regarding the level of implementation of GPP in the construction sector. Kozik et al. (2016) [33] in Poland claimed that the level of implementation of solutions for green public procurement in Polish public construction is mediocre and do not correspond to the content of the strategic documents and the need for pro-environmental public spending. 

Testa et al. [24] in their study on GPP in Italy concluded that 19% of public procurement used green specifications as award criteria. If the analysis is focused only on the most economically advantageous tender (MEAT) 87% of the procedures included green criteria and the average weight given to these criteria was about 18%. The most common award criteria were the requirements related to energy performance with a percentage of 30% in the tenders—and with high value tenders more likely to include green criteria. The authors did not find any correlation between the size of the procurer and the greenness of tenders.

Fuentes-Bargues et al. [8] in a study on environmental criteria in Spanish public works analyseanalysed a sample of a hundred projects tendered between 2008 and 2011, and found that the use of environmental criteria was about 35% and the average weight was about 5.7 points. The most used criterion was the environmental plan (EP) also called the environmental action plan (EAP)—but such a plan needs a clear and complete definition of its content.

The use of environmental criteria within construction sector tenders in these last two studies is lower than the European average of 40% according to the study of Renda et al. in 2012 [57] regarding GPP in the EU27.

### 2.2. Research on GPP on Universities

Public universities are public authorities and subject to the same control mechanisms for contracts as in public procurement, but very few studies have been conducted on GPP in the context of universities.

Mansi et al. [18] researched the impact of the demographic characteristics of procurement professionals on sustainable procurement practices. The professionals in this study belonged to 39 Australian universities. For the environmental dimension of SP practices, conclusions from the study show that gender (rather than age) influences procurement professionals. Women professionals are positively associated with environmental procurement practices.

Bala et al. in 2006 [21] performed an analysis on green practices at the Autonomous University of Barcelona. They made five case studies: office material; recycled toner cartridges; vending machines (Fairtrade coffee); reusable glass bottles in vending machines; and catering services. The authors concluded that there is no regular pattern for predicting success when trying to implement a suppliers’ greening program in public universities, but contract specifications, process implementation, market characteristics, supply chain profiles, and supplier characteristics are factors that must be considered.

Pacheco-Blanco and Bastante-Ceca [19] examined sustainable consumption at Spanish public universities. The research was carried out from two perspectives: External, with an analysis of websites and their information; and internal, with a survey of environmental departmental managers. Significant findings were that only 11 of 51 (21.5%) Spanish public universities had a public procurement manual (and then only for certain budgetary headings) and that environmental criteria were included mainly on food and catering services, gardening products and services, and copying and graphics paper.

Other research that includes a brief analysis of GPP at universities is the study performed by Fuentes-Bargues et al. [8]. In a sample of 100 projects analyseanalysed, twelve of the 100 projects were tendered by Spanish universities. In eight of the twelve cases studied, purchasing managers use an environmental criterion with a weighting between 3 and 5 points, and in one of the procedures the weight was 7.5 out 100 points.

### 2.3. The Structure of Spanish Public Universities

The organisation of university education in Spain is established by Royal Decree 1393/2007, of 29 October, in accordance with the general guidelines of the European Higher Education Area (EHEA) [58].

**Figure 1:**
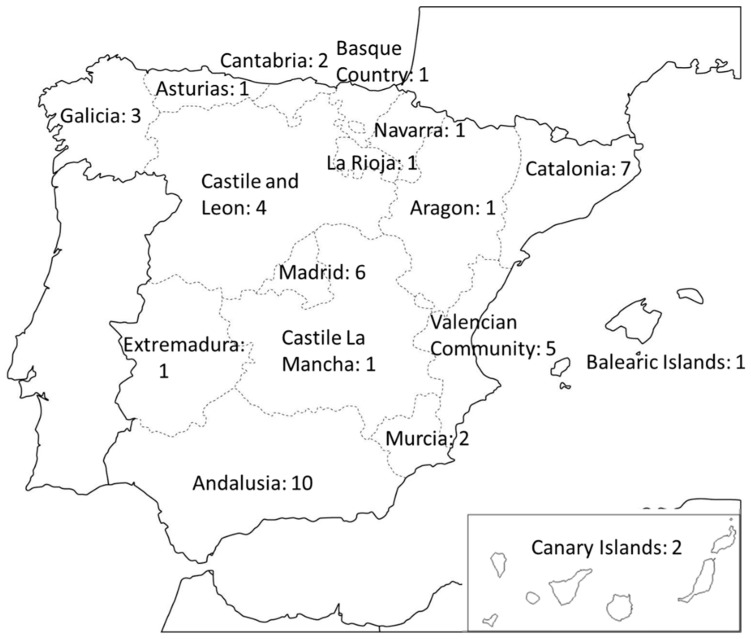
Distribution of Spanish public universities. Source: Authors.

The Spanish university system is made up of private and public universities, distributed throughout the country (Figure 1). There are 52 public universities and 32 private universities. Public universities are financed directly by the central state (National Distance University; Universidad Nacional a Distancia—UNED), or through the regional governments; and private universities are financed by private funds, student fees, and some public funds. 

In the academic year 2015–2016, 1,143,223 students were studying at public universities and 178,475 at private universities [59]. Universities offer official degrees (undergraduate, master, and doctoral) and university-specific degrees. The official degrees are designed by the universities in accordance with the general guidelines established by the state and must pass a verification and accreditation process. By contrast, the university-specific degrees are independently designed by each university.

Spanish public universities accounted for 0.9% of GDP in 2013 and 74% of this expenditure corresponded to labor costs [59], so the remaining 26% is related to products, services, and works—and thus justify the importance of studying universities as public procurement bodies.

## 3. Method

### 3.1. Measureament Technique: Content Analysis

The method used in this study is a content analysis of the documents obtained from calls for tenders. Content analysis can be defined as a method of observation where the researcher analyses the communication that a person has produced in order to understand their behaviour [60].

This research method is objective, systematic, quantifiable and generally applicable [61]. In this study, objective data from public tender documents published at web pages of the public administrations have been used, which allows the analysis to be reproduced by other researchers who wish to verify the results obtained. The contents have been analyseanalysed systematically, by obtaining data from specific parts of collected tender documents. 

The method is also characterized by the fact that it allows the results of the analysis to be quantified, either by the numerical nature of the data analyseanalysed or by their transformation and parameterisation into indicators and metrics useful for the interpretation of the results.

Content analysis has been used previously on research about GPP, as a single method or combined with other methods. Kippo-Edlund et al. (2005) [62] studied the uptake of green criteria by analysing the content of tenders in Sweden, Norway, Denmark, and Finland in order to verify the influence of environmental factors on award decisions. Bouwer et al. (2006) [39] investigated the uptake of GPP practices in the EU 25 through a content analysis of a large sample. Parikka-Alhola et al. (2006) [42] assessed tender calls and tender documents of 180 procedures published in 2005 in Denmark, Finland, and Sweden to characterize all the elements and criteria of the MEAT.

Palmujoki et al. (2010) [25] analyses 156 tender documents to study environmental criteria in the acquisition of goods and services in Sweden and Finland during two periods (2005 and 2007). Testa et al. (2016) [24] studied the degree of GPP in the construction sector in Italy by analysing content over a sample of 164 tenders collected from all Italy. Fuentes-Bargues et al. (2017) [8] studied the use of environmental criteria in public works over a sample of a hundred projects tendered in Spain.

In combination with other techniques, content analysis was used by Faith-Ell (2005) [63] in the study of the application of environmental requirements in the procurement process of Swedish road maintenance, and by Adham and Siwar (2012) [37] in the study of green public purchasing in the Malaysian ICT sector. 

### 3.2. Sample Selection

**Figure 2:**
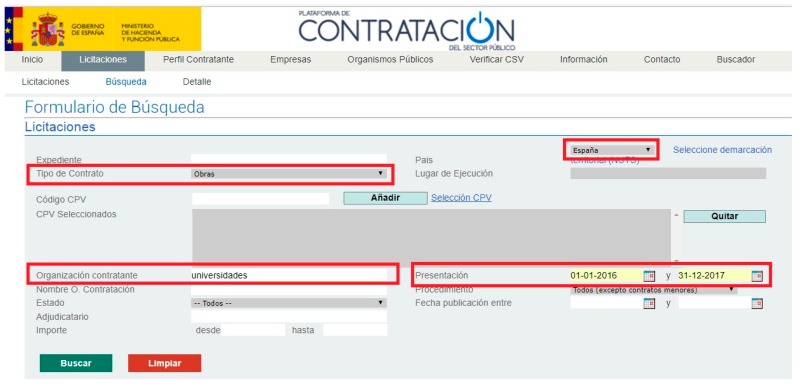
Spanish public sector procurement platform [64].

The tenders were collected from a Spanish government tender database (Plataforma de Contratación del Sector Público, in Spanish) (Figure 2) and completed from the university websites. The criteria for downloading the tenders were: Public authorities (universities); type of contract (works); and deadline for the tender (between the 1 January 2016 and 31 December 2017). Works tenders include, as defined by the European and Spanish legislation, the construction, maintenance, and renovation of buildings, roads, airports, in all public facilities. The dates were chosen to include the data for two full post-economic crisis years. The data collection process began in December 2017 and finished in March 2018. 

All the documentation available in the national database and the websites of the universities was downloaded. The main documents analysed were the administrative specifications, the tender notice, the technical documents, and the projects as complementary documentation. As in similar investigations, it was not possible to obtain all the documentation for the proceedings [8,24,65].

The method is divided into six steps. The first step is to study the project and the tendering documents. In step two, each sample case was analysed to locate any environmental criteria involved in the tenders. These environmental criteria are analysed and classified by subsector (civil engineering or building), geographical scope, and project budget. Thereafter, the weight of the environmental criteria is analysed and classified by subsector, geographical scope, and project budget. Finally, the environmental criteria identified is related with other criteria used in the tendering process. In the next step, a discussion and comparison with the results from other studies is included. Lastly, the conclusions are presented.

### 3.3. Characteristics of the Sample

A total of 361 procedures (N) were collected from the work tender calls of Spanish public universities in 2016 and 2017. When a procedure is divided into batches, these have been regarded as separate procedures. After a revision of the documentation, only 316 were useful for the analysis (N*). The distribution for universities is presented in the Table 1.

The files were classified according to various criteria. One of these was the year of the call: 120 were tendered for 2016 and 196 for 2017. 

The construction sector is divided into two subsectors. The building subsector includes all types of buildings: housing, factories, offices, schools, and sports facilities. Civil engineering work includes roads, ports, airports, railways, and water pipelines. In this case, within the building subsector, a division has been made according to the characteristics of the works, distinguishing between buildings and facilities (sanitation, air conditioning, electricity, etc.). Some 13.3% of the sample represents civil engineering works, 55% represents buildings, and 31.7% represents facilities.

Some 64% of the sample was composed of competitive tenders—and auctions formed the remaining 36%.

Five price levels were established according to the project budgets. The five levels were: Less than €200,000 (48.7%); between €200,000 and €1,000,000 (37.7%); between €1,000,001 and €5,000,000 (10.5%); between €5,000,001 and €10,000,000 (2.5%); and over €10,000,001 (0.6%). 

Under Spanish public procurement legislation there are several types of legal requirements that affect the time and complexity of the process, and a distinction is made between ordinary and urgent processes. In the sample, 93.7% were processed under the ordinary procedure and 6.3% using the urgent procedure. Another difference is between open procedures (all the firms reaching the requirements may participate) and negotiated procedures with, and without, advertising (only selected bidders can participate in such tenders, and the difference between with and without advertising is determined by the amount of the budget). Some 71.8% were tendered using open procedures, 8.5% by negotiated procedures with advertising, and 19.6% by negotiated procedures without advertising.

The common procurement vocabulary (CPV) code is a system for the identification and categorization of all economic activities that may be engaged in by means of public or competitive tender in the European Union [66]. These codes enable a classification to be made of the scope of the project. Table 2 shows the main CPVs and the frequency used in the sample of study. 

## 4. Results

The results obtained in the tendering analysis show that 19.3% of the projects studied include references to environmental criteria. If only the MEAT procedures are considered, then the percentage reaches 30.2%. The definitions and descriptions of the different environmental tendering criteria identified in the study sample are described in Table 3.

As can be seen in Table 3, the description of the different environmental criteria presents items that are very similar, and mainly related to the improvement of the energy efficiency of equipment, installations, and buildings. Environmental action plans (also termed within another criteria as environmental management plans) also appear as one of the main environmental criteria used and include the organisation of the environmental team, the actions to reduce and recycle the waste generated during the work, measures to reduce the use of fossil fuels, and the increased use of renewable energy.

The possession of EMAS (Eco-Management and Audit Scheme), ISO 14001, or similar certificates does not appear as an environmental tendering criterion. There are some references to preparing an environmental plan, or waste management plan, according to the ISO 14000 rules.

All of the environmental criteria described in Table 3 are value judgement criteria, rather than criteria valued through formulae. In some of the tendering documents appear partial scores for the required items that integrate environmental criteria—but none show the rating scale of the items.

The distribution of environmental criteria in the construction sector shows that 7 of the 61 tenders belong to the civil works subsector (11.5%), and 54 belong to the building subsector (88.5%). In the former subsector, the tenders with environmental criteria total 7 out of 42 tenders (i.e., 16.7%). In the building subsector, 54 of 274 tenders had environmental criteria (i.e., 19.7%), which reveals that the use of environmental criteria is almost the same in both subsectors. 

Table 4 identifies the universities that have used environmental criteria in the study tenders, the number of times, and the percentage regarding the total tenders of the university identified in the sample study. Some universities (Alicante, Jaen, and Polytechnic of Cartagena) use environmental criteria in more than 90 per cent of their contracting procedures. 

From the point of view of the contract execution budget (CEB), environmental criteria are included in: 12.3% of the projects with a CEB of less €200,000; 23.5% of the projects with a CEB between €200,001–€1,000,000; 33.3% of the projects with a CEB between €1,000,001–€5,000,000; 12.5% of the projects with a CEB between €5,000,001–€10,000,000; and 100% with a CEB over €10,000,000. 

Table 5 shows that tenders with environmental criteria are related with the type of administrative processing of the procedure, and with the manner that bidders participate in the tender. Results show environmental criteria are hardly ever used in negotiated procedures and in urgent tenders.

Tenders with environmental criteria have been classified according to the CPVs. Code 45000000 shows 14 procedures with environmental criteria (42.4% of the total procedures with this code); Code 45214400 with 6 procedures (21.2%); and Code 45214000 with 4 procedures (14.8%). These are the main significant CPVs with environmental criteria, but no relationship can be established between specified CPVs and environmental criteria.

The weighting of the environmental criteria in the sample was studied. The average weight of the environmental criteria in the 61 works of the sample is 6.5 points over a hundred. The maximum weight of the environmental criteria was 37 points over 100. In a project at the University of Valladolid (construction of an R + D + i and specialized training building at the Duques de Soria Campus) some environmental criteria were used. In this tender, one criterion had a weight of 19 points and consisted of measures to improve the general sustainability of the building, improve the environmental conditions, and obtain the highest possible LEED and VERDE rating. The most often used weighting is ‘5 points’ (occurring on 29 occasions).

**Figure 3:**
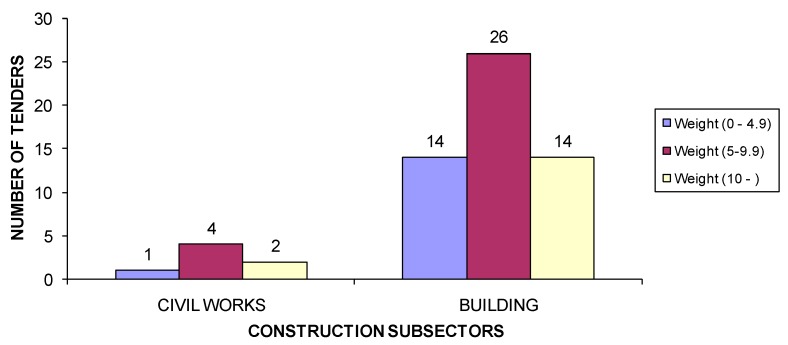
Number of tenders according to weighting of environmental criteria by construction subsector.

If a comparison is made between the weighting of the environmental criteria and the subsector (Figure 3), then the most used weighting range for environmental criteria was between 5 and 9.9 points over 100 (in both civil engineering and building subsectors).

**Figure 4:**
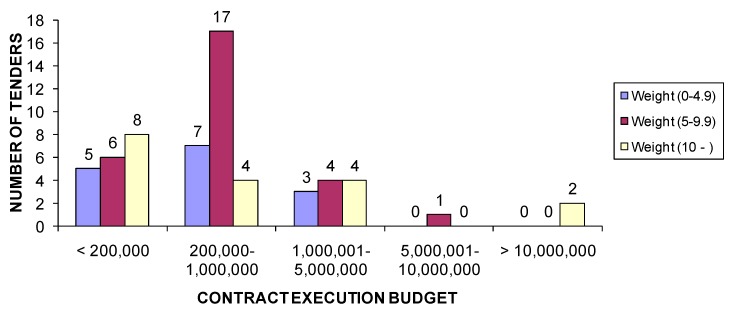
Number of tenders according to environmental criteria weighting by contract size (in euros).

In Figure 4 a comparison is made between the weight of the environmental criteria and the CEB, and the results show that the most used weighting range for environmental criteria is between 5 and 9.9 points for projects with a CEB between €200,000 and €1,000,000. For all other bandings, there are few projects and no substantial difference between weights. 

Finally, the relationship between environmental criteria and other criteria used in the tendering process was studied. In the 61 projects identified with environmental criteria, the main tendering criteria was: price (all projects); work programme (42 of 61 projects); enhancement of the guarantee period (27 of 61 projects); description of the construction process (26 of 61 projects); and enhancements offered (23 of 61 projects). Criteria such as quality systems and health and safety procedures were used in fewer procedures. No relationship can be established between the weight of the environmental criterion (6.7%) and the weight of other criteria; but the relative importance of the environmental criteria in the 61 procedures can be indicated. Price is the criterion with the greatest weight (55.5%), followed by work programme (24.6%), description of the construction process (18.1%), enhancements (10.6%), and completion time (8.8%). Environmental criteria have similar weights to the guarantee period (6.8%) and quality systems (6.6%)—and higher weights than health and safety procedures (4.1%).

## 5. Discussion

In the study sample, 19.3% of the Spanish public universities use environmental criteria when contracting construction projects. This value is lower than the 40% average in the EU27 in 2012 as defined by Renda et al. [57]; and is also lower than a recent study made in Spain from a sample of a hundred projects and in which 35% included environmental criteria [8]. However, the percentage is equal to a study performed by Testa et al. with tendered works between 2012 and 2013 in Italy [24], where 19% of the procedures included green specifications as award criteria; and similar to another study conducted in Italy in 2010, also by Testa et al. [44], with 23% of procedures. There are some explanations for these differences. Firstly, there is a clear difference between Mediterranean countries and the ‘Green 7’ (Sweden, Germany, Austria, Denmark, United Kingdom, Netherlands and Finland) [39,57]. Secondly, and in agreement with Pacheco-Blanco et al. [19] and Bala et al. [21], there is still a long way to go to improve the environmental dimension within university procurement. Thirdly, and as previously indicated by Testa et al. [24], results obtained from the analysis of the call for tenders are more conservative than the results obtained by surveys of public administration managers.

Table 3 shows the description of the environmental criteria used in the contracts in the sample. All the criteria are assessed with value judgment criteria and the criterion most often used is ‘energy efficiency and sustainability improvements’. This criterion is aligned to the main criteria indicated by the European Commission guide on GPP [53] and is in concordance with the research of Testa et al. [24] in Italy (in this case, the main criterion was energy performance). This criterion reflects the numbers construction projects tendered by Spanish public universities (mainly buildings, building installations, and space conditioning for parking or sports annexes). 

Enhanced aspects, as defined by Commission European Guides on GPP [52,53] and/or described in previous research [54,56], such as life cycle assessment (LCA), or carbon footprint (CF), do not appear in the environmental criteria for working contracts, even if there are references within other criteria, such as the LCA, or characteristics of the materials used, both in buildings and civil engineering works.

Environmental plans (EPs) or environmental action plans continue as one of the main environmental criteria and, in the same way as in the study of Fuentes-Bargues et al. [8], a standardization and/or a guide for the elaboration of the EP is necessary. Several items are repeatedly integrated into EPs, such as preventive and corrective project measures, the use of recycled and reused materials, and the environmental procedures of the tendering company, promoting the relationship with the EMS, ISO 14001, or equivalent certification systems used by the company. 

EMS, ISO 14001, or similar certification systems, are not used directly as award criteria, in compliance with European directives [26,27] and Spanish legislation [28,29,51], but are used as a part of other criteria, or for coordinating environmental measures for working with the environmental systems of companies (Table 3), since they are essential for encouraging good environmental practices in companies and achieving a sustainable construction systems [7].

As mentioned above, the intrinsic characteristics of the universities mean that public works belong mainly to the building subsector—but the percentage of environmental criteria in this subsector is only slightly higher than the civil engineering subsector, so it can be aligned with previous research where it was affirmed that the use of environmental criteria is more common in civil engineering works than in building works [8].

No conclusions on the use of environmental criteria can be associated with the geographical distribution of the universities. It can simply be said that there is no homogeneity between the behaviour of universities. There are some that do not use environmental criteria and others that make considerable use of them. This result is in line with that obtained by Pacheco-Blanco and Bastante-Ceca [19] regarding environmental performance in the tendering of products and services in the study of Spanish universities.

Some aspects of the contracting process have been studied to determine if these influence the use of environmental criteria, and it can be affirmed that the CPV code, the type of administrative processing, and the method of participation have no influence. It can be concluded that environmental criteria is hardly ever used in negotiated procedures and in urgent processes.

The results of the study show how the use of environmental criteria is widespread in projects with budgets above €10,000,000 (100%), and between 25% and 34% in those projects with budgets between €200,000 and €5,000,000. Such criteria are less used in projects with budgets below €200,000 (12.3%). Therefore, depending on the complexity and/or the size of the project, a certain relationship between project budget and the use of environmental criteria can be established.

Regarding the importance of environmental criteria within the contracting process, it must be pointed out that the average weighting for such criterion (compared to the total tender) in this study is 6.5 points out of 100, slightly higher than the 3–5 points obtained by Palmujoki et al. [25] in their research in Sweden and Finland, as well as the 3.3 points obtained by Nissinen et al. [41] in Sweden, Finland and Denmark. This weight is similar to the results obtained by Igarashi et al. [51] in Norway, Fuentes-Bargues et al. in Spain [8], and Testa et al. in Italy [24].

Environmental criteria are fifth in importance in the study sample, after price, work program, the description of the construction process, any enhancements in the project, and delivery time. It has the same or greater importance than the guarantee period, quality systems, and health and safety procedures. This level of importance is in concordance with previous studies [8,51], but with slightly better results for relative weights. This data confirms affirmations in other studies about the little importance attached to the environmental criteria by public administration managers and technicians [7,8,24].

## 6. Conclusions

GPP is an important topic for sustainable development that should be further developed and whose future implementation needs to be better defined. The research results could help public authorities related to the public construction sector, such as Spanish public universities.

The results of this study show that the use of environmental criteria in the works tendered by Spanish public universities is low (19.2%) in comparison with the results of other studies in local, regional, and national administrations in Spain and other European countries. It is necessary to encourage GPP practices in the contracting process in order to comply with the environmental policies that various universities have defined as part of their institutional policies. As part of the encouragement of GPP practices, plans for environmental training of the administration’s technical staff in public universities should be included.

The main environmental criterion used in the public procurement of works at Spanish public universities is improvements in the energy efficiency of the equipment, installations, and buildings; however, a formula or objective approach for evaluation it is undefined. The other environmental criteria identified in the research are assessed by value judgement, so it is necessary to develop indicators or formulae for environmental items to improve the relationship of the criteria with the object of the project and so facilitate an objective assessment of the criteria.

The use of environmental criteria in tenders by Spanish public universities is similar in both the building and civil engineering sub-sectors, and more used in projects with higher budgets. This cannot be associated with the staff, nor the size of the universities; and can only be associated with the complexity and size of the projects, environmentally and/or economically.

The average weighting of the environmental criteria was found to be low (6.5 points out of 100): fifth in importance in the sample after price, work programme, the description of the construction process, project enhancements, and delivery time—but with the same or even greater importance than the guarantee period, quality systems, and health and safety procedures.

This research can be extended to other types of Spanish public administrations and/or other countries to discover the current status of GPP applications and provide starting points for the development of tools that encourage sustainable public procurement and valuation tools for the assessment of the environmental criteria in the contracting process.

## Figures and Tables

**Table 1:** Number and provenance of the procedures of the study sample.

University	Region	*N*	N*
University of Almeria	Andalusia	0	0
University of Cadiz		6	6
University of Cordoba		5	5
University of Granada		6	6
University of Huelva		4	4
International University of de Andalusia		0	0
University of Jaen		10	10
University of Malaga		10	8
University Pablo of Olavide		0	0
University of Sevilla		22	22
University of Zaragoza	Aragon	5	5
University of Oviedo	Asturias	2	2
University of Basque Country	Basque Country	16	16
University of La Laguna	Canary Islands	9	8
University of Las Palmas of Gran Canaria		2	2
University of Cantabria	Cantabria	2	2
International University of Menendez Pelayo		0	0
University of Castile La Mancha	Castile La Mancha	4	4
University of Burgos	Castile and Leon	5	5
University of Leon		13	11
University of Salamanca		22	22
University of Valladolid		23	7
Autonomous University of Barcelona	Catalonia	14	12
University of Barcelona		0	0
University of Girona		5	3
University of Lleida		4	3
University Pompeu Fabra		4	4
University Rovira i Virgill		3	3
Polytechnic University of Catalonia		7	7
University of Alcalá	Madrid	8	8
Autonomous University of Madrid		13	13
University of Carlos III		7	7
University of Complutense of Madrid		28	28
National University of Distance Education		2	2
University of King Juan Carlos I		3	3
Polytechnic University of Cartagena	Murcia	1	1
University of Murcia		23	23
Public University of Navarre	Navarre	2	2
University of Alicante	Valencian Community	14	14
University Jaume I		4	0
University of Miguel Hernandez of Elche		5	5
Polytechnic University of Valencia		5	5
University of Valencia		9	4
University of Extremadura	Extremadura	10	2
University of A Coruña	Galicia	2	2
University of Santiago of Compostela		9	7
University of Vigo		9	9
University of Balearic Islands	Balearic Islands	2	2
University of La Rioja	La Rioja	1	1
	TOTAL	361	316

*N* are the number of procedures collected from the call for tenders; N* are the number of procedures that present all the documentation for the analysis.

**Table 2:** CPV’s of the sample of study.

CPV	Frequency	Description
45,000,000	33	Construction work
45,214,400	33	Construction work for university buildings
45,214,000	27	Construction work for buildings relating to education and research
45,200,000	16	Works for complete or part construction and civil engineering work
45,261,900	14	Roof repair and maintenance work
45,330,000	14	Plumbing and sanitary works
45,343,200	14	Firefighting equipment installation work
45,300,000	12	Building installation work
45,210,000	11	Building construction work
45,310,000	11	Electrical installation work

**Table 3:** Environmental criteria and its description in the study sample.

Description of the Environmental Criterion	Frequency That Environmental Criteria (EC) Mentioned	Description
Energy efficiency and sustainability improvements	13	Improvements related to the energy and environmental efficiency of the equipment installed and which result in a reduction in electrical consumption, and providing an improvement in the performance of the new installation.
Quality control plan and environmental and quality measures	9	Without description.
Environmental Action Plan	7	If the work is carried out within an environmental management framework using an EMAS, ISO 14001, or equivalent certification system. If the materials to be used by the tenderer comes from a recycling or reuse process. Whether the materials to be used by the tenderer can be reused or recycled. These materials must comply with technical specifications. If the execution of the work includes any of the following measures: environmental management of land and building materials; landscape restoration; reduction in the generation of waste; reduction in pollution by discharges, noise, air or soil; reduction of visual, cultural, or sociological impact; limitation of impact on local fauna and flora; reduction in the use of fossil fuels or use of renewable energy.
Quality management, prevention, environment and energy efficiency	4	Environmental management plan to be drawn up by the employer, or the joint venture, if awarded as a contract. Environmental organisation chart proposed by the employer, or the joint venture, for the execution of the work and that of the technicians working in environmental management, with a description of the environmental responsibilities for each of the positions.
Technical improvements related to project	3	The improvements will be justified as a priority in terms of the increase in the building’s performance in terms of obtaining the highest LEED and VERDE rating, the general sustainability of the building, and any improvement in the environmental conditions.
Quality and environmental control	3	The contribution of an environmental management system will be assessed, including the technical and economic resources that the bidder intends to use for this purpose.
Environmental management	3	Adequacy and development of the waste management plan. Environmental report for the work.
Sustainability	3	The measures proposed by the company that involve improvements related to protecting the environment will be assessed. Improvements may be made during the construction process of the building (or to solutions or features that will be integrated into the final result) that will make the building more sustainable and environmentally-friendly. The measures to be considered include those that improve the energy consumption of the building, those that lead to savings in water consumption, and the use of recycled materials or materials that are easily recoverable or reusable at the end of their useful life. It will also be favourably assessed if the wood, forest products, or processed products derived from wood, or other forest products that the company use in the works, have an International FSC (Forest Stewardship Council) or PEFC (Programme for the Endorsement of Forest Certification Schemes) certificate, or any other internationally recognised certificate confirming that the wood comes from sustainably managed forests.
Environmental actions	2	An identification of the work units and operations that may generate environmental impacts, and therefore require monitoring. An environmental vigilance plan must also establish a waste management and pollution control system, according to ISO 14000 or similar, as well as the appropriate preventive and corrective measures.
Improvements in the field of energy efficiency	2	Proposals that are considered an enhancement of the energy efficiency of the projected installation and without additional cost will be considered.
Improvements to reduce environmental impact	2	Replacement of conventional plastic paint with ecological paint: if petroleum or derivatives are used in the manufacturing process, these are to be replaced with components strictly of mineral origin (silicates, calcium, or clays), or vegetable origin (resins, oils or waxes). Replacement of concrete in the project specifications by concrete made with recycled aggregates. Use of products with recycled plastics in pipes and conduits (as permitted).

**Table 4:** Distribution of environmental criteria by universities.

University	Number of Times Environmental Criteria Used (EC)	EC/N* (%)
University of Alicante	13	92.9
University of Jaen	9	90
University of Malaga	7	87.5
Polytechnic University of Catalonia	5	71.4
Polytechnic University of Valencia	4	80
University of Granada	4	66.7
Autonomous University of Barcelona	4	33.3
University of Valencia	3	75
University of Castile La Mancha	3	75
University of Valladolid	3	42.9
University of Murcia	2	8.7
University of Lleida	1	33.3
University RoviraiVirgill	1	33.3
Polytechnic University of Cartagena	1	100
University of Seville	1	4.5

N* are the number of procedures that present all the documentation for the analysis.

**Table 5:** Environmental criteria according to the type of administrative processing and participation approach.

**Type of Administrative Processing**	**Tenders with Environmental Criteria**	**Total Tenders**
Ordinary	59	296
Urgent	2	20
**Method of Participation**	**Tenders with Environmental Criteria**	**Total Tenders**
Opened	50	227
Negotiated with advertising	8	27
Negotiated without advertising	3	62

## Abbreviations

AHP	analytic hierarchy process
ANP	analytic network process
AUB	Autonomous University of Barcelona
CEB	contract execution budget
CF	carbon footprint
CPV	common procurement vocabulary
DBFM	design-build-finance-maintain
EAP	environmental action plan
EHEA	European Higher Education Area
EMAS	eco-management and audit scheme
EMS	environmental management systems
EPD	environmental product declarations
EP	environmental plan
FSC	Forest Stewardship Council
GDP	gross domestic product
GPP	green public procurement
ICT	information and communication technology
LCA	life cycle assessment
MEAT	most economically advantageous tender
OECD	Organization for Economic Co-operation and Development
PEFC	Programme for the Endorsement of Forest Certification Schemes
SCP	sustainable consumption and production
SPP	sustainable public procurement
TED	tenders electronic daily
UNED	Universidad Nacional a Distancia
WSSD	World Summit on Sustainable Development
